# Brain Matter and Mind Action

**Published:** 1869-01

**Authors:** 


BRAIN MATTER AND MIND ACTION.—We have received an interesting paper with the above title from Prof. A. J. Steele of St. Louis, Humbolt Medical College, formerly graduate of Buffalo Medical College, and for a time Demonstrator of Anatomy in this institution. Dr. Steele was also House Physician to the Buffalo General Hospital where he won for himself many friends. His paper shows him as formerly, an earnest worker. It consists of cases illustrative of the proposition, that loss of brain-substance will be followed by impaired cerebration, the opposite view having been generally entertained, viz.: that loss of brain matter could be endured to a very considerable extent without impairment of the mental faculties. Many cases are related showing the correctness of the doctor's proposition, and even the cases cited as proving that impaired mind might not follow loss of brain substance are followed out and more carefully noted and finally prove exactly the reverse. The celebrated Tamping-rod case in Vermont, reported by Prof. Gross as recovering without loss of mental power turns out as reported by his attending surgeon, Dr. Harlow, to have been very much impaired.

The paper is highly interesting and instructive, and so far as cases cited, his proposition is fully sustained.

